# Food Consumption Structure and Food Security—Through Mediating Effect Analysis of Agricultural R&D and Agricultural Investment

**DOI:** 10.3390/ijerph191912524

**Published:** 2022-09-30

**Authors:** Wentai Bi, Yu Song, Yang Liu, Zongze Li, Ying Zhang

**Affiliations:** 1 College of Economics and Management, Henan Agricultural University, Zhengzhou 450046, China; 2 Faculty of Education, University of Malaya, Jalan Universiti, Lembah Pantai, Kuala Lumpur 50603, Malaysia

**Keywords:** food consumption structure, food security, food security evaluation system, agricultural investment, agricultural R&D

## Abstract

Food security is the cornerstone that ensures the stable development of a country. Based on panel data of 31 provinces (including autonomous regions and municipalities) in China from 2015 to 2019, we use the mediating effect model to explore the mechanism by which food consumption structure affects food security. The results indicate that grain consumption has a significant promoting effect on food security, while plant and animal food consumption have significant inhibiting effects on food security. Furthermore, agricultural R&D and investment play mediating roles in the impact of food consumption structure on food security. Obvious differences exist in the relationship between food consumption structure and food security between urban and rural areas, as well as between Eastern, Central, and Western regions. Animal food consumption had a negative and significant impact on food security, with a stronger effect on rural residents than on urban residents. Compared with the central and western regions, grain consumption and animal food consumption in the eastern region had a stronger marginal impact on food security. This paper enriches and expands the research on influencing factors of food security from the perspective of consumer demand, which has important theoretical value and practical significance for ensuring food security.

## 1. Introduction

Food security provides an important foundation for ensuring social stability, high-quality agriculture, and sustainable agricultural development, and the tension relating to ensuring food security cannot be eased [1]. From the perspective of total grain supply, however, China’s grain production has entered a new period of development, with total grain output in 2021 stabilizing at 1.33 trillion tons, maintaining an increase in production for 18 consecutive years. Exceeding the international standard quota in per capita share of grain, under the double impact of the increasingly tense international trade environment and the COVID-19 pandemic, the original international grain supply chain has been hindered, increasing the imbalance and instability of food supply. From the perspective of the grain supply structure, the main contradiction between domestic food consumption and grain production has shifted from the past, when the total amount of grain could not meet the needs of the residents, to the present difficulties associated with adapting to the upgrading of the food consumption structure, in terms of grain types and quality [2]. Therefore, it is necessary to explore the influencing factors of food security under the new development pattern, which is more conducive to ensuring food security.

As the most populous country in the world, China’s food security is not only an urgent matter for its economic development and social stability, but also has great significance for global food security [3]. China’s food security state remains ambiguous owing to rapid population and economic growth. Previous studies have measured food security in China through food supply and demand [4]. One is from the perspective of supply, including land policy and technological progress. Labor migration, land circulation, capital investment, and rural non-point source pollution have significant impacts on agro-ecological security and sustainable food development [5]. The choice of technological progress path also affects food security, and the impact of technological progress on unskilled labor enhancement is obvious [6]. The second path is from the perspective of demand for food consumption. Compared with research from the supply perspective, the literature from the demand perspective is relatively limited, mainly including the prediction of future food consumption demand, the impact of grain prices on food consumption, and the evolution and influencing factors of the spatio-temporal patterns of grain consumption [7].

In recent years, Chinese food consumption concepts, behaviors, and patterns have been changing continuously, and the structure of domestic food consumption has become an important part of the demand structure [8]. The improvement of consumption quality brought about by changes in the food consumption concept and the pursuit of green, healthy, and personalized directions have become a new food consumption trend [9]. Zheng et al. [10], stated that the evolutionary trajectory of the food consumption pattern of Chinese residents is basically in line with Engel’s Law, and with the growth of income, the future diet structure will be converted to a coordinated model of plant and animal food ratios. The transformation of the food consumption structure has affected agricultural carbon emissions, the grain cultivation behaviors of farmers, and agricultural production adjustments [11,12]. Timmer [13] stated that the transformation of grain consumption in China has reduced the proportion of grain consumption in total food consumption, therefore, it is necessary to establish a new concept of food security.

In summary, the existing literature on the influencing factors of food security has mostly focused on the supply perspective, and there have been few studies on food security from the demand perspective. The research on food consumption structure and food security is mostly qualitative research, and some authors believe that changes in consumption structure lead to an increase in the amount of food consumption, thus hindering food security. Based on Marxist production and consumption theory and using panel data from 31 provinces (including autonomous regions and municipalities) in China from 2015 to 2019, we aim to empirically test the impact effect and mechanism of food consumption structure on food security.

The rest of this paper is organized as follows: First, the theoretical basis and literature review are based on the construction of the hypothetical model. Next, we introduce the methodology and present the descriptive statistics of our data. Then, we report and discuss our estimation results. Finally, conclusions and policy implications.

## 2. Literature Review and Research Hypotheses

### 2.1. Food Consumption Structure and Food Security

Changes and trends in food consumption are an important part of food security, as well as an important basis for improving the food supply and demand system, formulating food policies, and adjusting the agricultural structure. Food security needs to take into account three levels: the macro level, to ensure the total production of food; the mesoscopic level, to enhance the ability to regulate and control food between regions; and the micro level, to ensure food availability for low-income households [14,15]. Therefore, the core link of food security is to ensure an adequate and stable supply of food, achieving a balance between supply and demand. The academic community at home and abroad has conducted extensive research on food security evaluation methods, and Table 1 lists the representative food security evaluation systems. Standard conceptual frameworks of food security, usually associated with the Food and Agriculture Organization, have four pillars: availability, access, utilization, and stability [16]. This definition has been critiqued and refined [17,18]. Availability corresponds broadly to the food supply. Access refers to effective demand for food and proximity to markets. Utilization is about the biological processing of food, which is partly related to dietary quality. Stability captures the dynamic aspect—as food is a daily necessity, being food secure requires stability in the other three pillars over time [19,20]. Allee uses 34 unique indicators to cover broad aspects of food security from average food supply, to diet diversification, to the presence of a formal grocery sector. The indicators are organized into three categories (Affordability, Availability, and Quality/Safety) [21]. Some scholars believe that food security is affected by such factors as agricultural production, food price volatility, the proportion of food disasters, national production, or the country’s import or exchange capability [22]. Concurrently, Schindler et al. [23], considered not only the basic supply of food, but also the purchasing power of households, food security, economic and political stability, and international trade.

Changing food preferences could be an important demand shifter in the twenty-first century [31]. Various factors could influence food consumption structure, such as income growth, the progress of urbanization, structural changes in population demographics, health and environmental concerns, and retail market transformation [32]. Food consumption structure refers to the variety, quantity, and proportion of various types of food in the diet. According to the definition of food consumption structure, this paper draws on the research of Zhang et al. [33], measuring the total food consumption with respect to the proportion of grain consumption, plant food consumption, and animal food consumption. From the perspective of food demand, the structure of food consumption has changed, and the food security situation has become more severe. With the rapid growth of China’s economy, the living standards of residents have been greatly improved, the amount of grain consumption has decreased, the consumption of animal food has increased, and the level of consumption has changed from ‘adequate and ample’ to ‘nutritive and healthy’. The change in the structure of food consumption has led to an increase in processed foods and meat products. The expansion of the scale of production in the grain-consuming animal husbandry industry has stimulated the growth of demand for deep-processed grains and feed grains. The per-capita feed grain and industrial grain have risen from 119.41 kg in 2001 to 268.65 kg in 2020, which has brought regional equilibrium changes in food supply and demand, and the problem of structural imbalance in grain has become prominent.

Changes in food demand will also lead to changes in the rationing of food supply resources, and the relationship between food consumption structure and food security may be complex. Marxist production and consumption theory holds that production and consumption are unified as opposites and influence each other. First, food consumption affects the structure of grain production. A country’s food consumption is influenced by factors such as its income distribution level, population structure, eating habits, and topographic characteristics, which determine the country’s food production decisions, to a certain extent [34]. Second, food consumption affects the scale of grain production and the operation of the production cycle. If the product cannot be sold, the production cycle is destroyed, and the scale of reproduction is bound to decrease. Based on the positive incentive effect, when the changes in demand and supply structures fail to match each other, the demand structure is bound to cause adjustment of the supply structure, leading to unbalanced growth between industries [10]. The impact of food consumption on food security is manifested in the fact that, under the role of “economic rationality,” decision-making criteria, and market mechanisms, rational production and operation entities spontaneously and scientifically allocate production factors and rationally utilize endowment resources, such that the allocation of production factors such as land scale, agricultural science and technology, management mode, human capital, and economic systems tend to be reasonable, in order to achieve the best operational benefits in agriculture [35]. An adjustment of the dietary structure of residents triggers the transformation of crop production. When the productivity matches the production relationship and the supply and demand pattern is optimized and adjusted, the scale of grain production may be expanded, thus promoting the improvement of production efficiency and ensuring food security. However, plant food consumption and animal food consumption will squeeze the space of the planting industry to a certain extent, reduce the yield of food crops, and endanger food security [8].

Based on the classification of food consumption structure and the above discussions, the following three competitive hypotheses are proposed:

**Hypothesis** **1** **(H1).** *Grain consumption has a positive effect on food security.*

**Hypothesis** **2** **(H2).** *Plant food consumption has a negative inhibitory effect on food security.*

**Hypothesis** **3** **(H3).** *Animal food consumption has a negative inhibitory effect on food security.*

### 2.2. Food Consumption Structure, Agricultural R&D, and Food Security

Consumption has positive externalities for production and promotes investment in technological research and development. First, consumer demand is the source of technological innovation and innovation for producers. Consumers pass demand information to producers through purchasing behaviors, and producers adopt active research and development strategies to meet consumer demand and achieve their own profitability. Second, consumer demand for innovative products objectively promotes product improvement. Guided by the motivation to pursue profits, producers pay more attention to the experience of consumer product utilization and the technological contents of products. Production determines consumption and without production there is no consumption. From a dynamic point of view, consumption reacts to production, and the consumer-led consumption structure is upgraded through the effects of new product consumption on R&D. As an important part of consumption, the food consumption structure provides important guidance for the adjustment of agricultural factors, thus affecting agricultural R&D. The impact of agricultural R&D on food production and security is embodied in the following aspects: First, agricultural R&D promotes the formation of scale effects in grain enterprises. As a key link in promoting the quality and competitiveness of R&D in the grain industry, scientific and technological progress forms a market effect under the condition of increasing the degree of use of R&D results and reducing the average cost of R&D, ultimately realizing an increase in the scale effect of grain enterprises and promoting food security. Second, agricultural R&D helps the food industry to form an agglomeration effect. Patents, information, and technology are shared, and different business entities can imitate each other’s innovations to reduce R&D and production costs, thus realizing the agglomeration effect and the circular accumulation of R&D activities [31]. Based on the above, the following hypothesis is proposed:

**Hypothesis** **4** **(H4).** *The structure of food consumption affects food security by influencing agricultural R&D, which plays an intermediary role between food consumption structure and food security.*

### 2.3. Food Consumption Structure, Agricultural Investment, and Food Security

Consumption drives investment demand and promotes the expansion of production scale. The diversification of consumer demand and the upgrading of structure promote the vertical development of resource input and related service industries [34]. Based on the Marxist production and consumption theory, consumption has the role of “leading” in influencing social demand, by reacting to production and expanding the economic form of reproduction, mainly referring to the investment in production and means of subsistence. Consumption on the demand side directly affects the production expectations and arrangements on the supply side, and the relevant investment and financing activities will also change accordingly. Consumption drives investment, investment drives production, and the transformation of food consumption structure will also have an impact on investment decision-making behavior and industrial structure in the market. Based on the theory of factors of production, agricultural investment is an important factor in promoting agricultural growth and ensuring food security [36]. On one hand, agricultural fixed investments improve infrastructure, agricultural technology and equipment, transportation networks, and agricultural production conditions, thus promoting the allocation of grain industry resources and achieving high-quality development of the grain industry. Investment in agricultural fixed assets has driven large-scale operations and the “grainization” of planting structures in the region, improving production efficiency and grain outputs while promoting food security. On the other hand, the increase in grain production effect prompts relevant stakeholders to obtain more benefits, arouses more interest and attention, induces a new round of agricultural investment, and ultimately forms a benign interaction between the improvement of comprehensive grain production capacity, thus ensuring food security. Based on the above, the following hypothesis is proposed.

**Hypothesis** **5** **(H5).** *The structure of food consumption affects food security by influencing agricultural investment, which plays an intermediary role in the relationship between food consumption structure and food security.*

**Figure 1:**
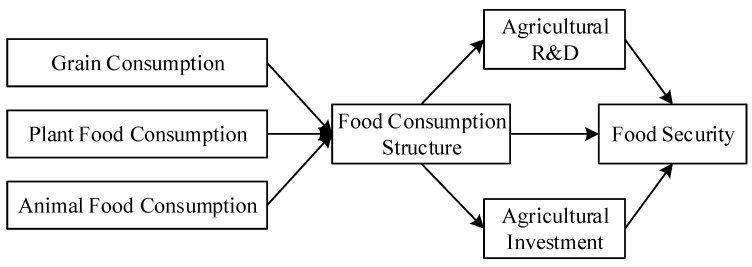
The overall theoretical framework diagram.

Therefore, the food consumption structure may indirectly affect food security through the two paths of agricultural R&D and agricultural investment. In conclusion, the influence mechanism of food consumption structure on food security and the role path of agricultural R&D and agricultural investment are shown in Figure 1.

## 3. Empirical Methods and Data Description

### 3.1. Data Sources and Variables

**Figure 2:**
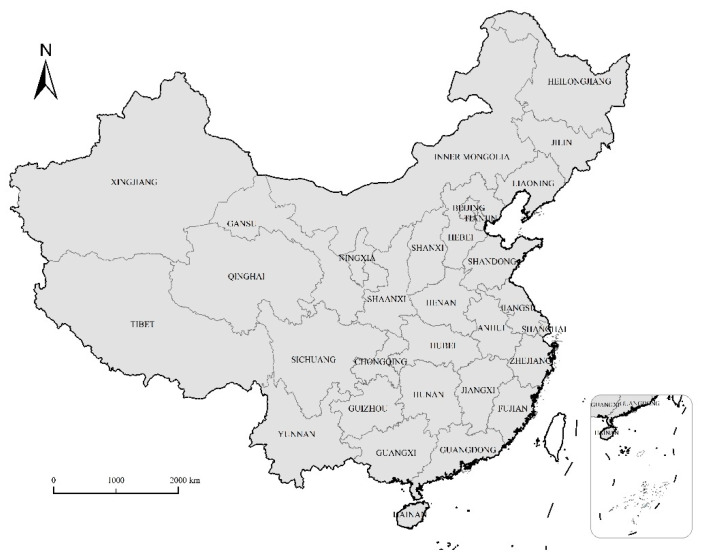
Spatial distribution of the study area.

The food consumption and food security data of residents in 31 provinces in China (excluding Hong Kong, Macao, and Taiwan, which are not included in the scope of the study due to the inaccessibility of data) from 2015 to 2019 were selected as samples. The location map of the study area is shown in Figure 2. We chose 2015 as the starting year due to incomplete reports on the main food consumption of residents in various regions in the years prior to 2015. Data on the structure of food consumption, agricultural investment, and control variables were derived from the China Statistical Yearbook; agricultural R&D data were derived from the China national knowledge infrastructure patent database, and the indicators of the food security evaluation index system were manually compiled from the data of the China Rural Statistics Yearbook. In order to avoid the adverse effects of missing data, some missing data were supplemented by interpolation. In order to eliminate the influence of outliers on the accuracy of the estimated results, Winsorization was performed for all continuous variables in the proportion of the highest and lowest 1%. Finally, a total of 155 observations in 31 provinces from 2015 to 2019 were obtained.

(1) Dependent variable. On the basis of the above-mentioned food security evaluation index systems in Table 1, combined with the definition of food security and China’s national conditions, we established a novel food security evaluation index system [27]. Specifically, the developed system includes 3 first-level indicators—namely, availability, stability, and sustainability—and 10 second-level indicators. The rationale for choosing these indicators is as follows. Considering availability, if the impact of international trade on China’s food security is not taken into account, the core problem of food security is the problem of food supply. The availability dimension includes “grain production per unit area”, which is used to measure the agricultural production capacity of the province; “grain sown area”, which is the basic condition of grain supply—to ensure the stability of grain production, we must first ensure the stability of the grain sown area [29]; “agricultural machinery power” is an important embodiment of the country’s agricultural modernization [34]; and, as for “the amount of fiscal support for agriculture”, the government’s stable investment in agriculture provides an important guarantee for food security. The FAO’s original definition of food security was that families and individuals could buy enough food at any time, suggesting that food security is true only when people’s basic food ration needs are met. As such, this index is measured in terms of “food share per capita” [29,37]. Considering Stability, the stable production of grain and effective supply is one of the goals of our food policy. This is measured using two secondary indicators: “fluctuation coefficient of food production” and “proportion of food affected”. Considering Sustainability, sustainable food development is an important means to cope with extreme weather and ecological deterioration, thus ensuring food security. It is measured using three secondary indicators: “pesticide application amount”, “fertilizer application amount”, and “plastic film use”. Although the excessive application of chemical fertilizers and pesticides can increase yields in a certain period of time, they may cause soil compaction and non-point source pollution, which is not conducive to the long-term stability of food production.

Following the existing literature, we used the coefficient of variation method to empower the indicators of the proposed food security index system [25,38]. The coefficient of variation method is a method by which weights can be obtained for factors, considering the degree of dispersion between variables and, thus, avoiding the arbitrariness of human subjective judgment. Due to the different dimensions, there was no direct comparison between the variables, and the coefficient of variation of each variable needed to be used to measure the degree of difference in the values of the indicators, in order to eliminate the influence of the dimension.

The coefficient of variation of each indicator and its corresponding weight formula were calculated as follows:

(1) $$V_{i}=\frac{\sigma_{i}}{\bar{x_{i}}}\left(i=1,2,\ldots ,n\right)$$

(2) $$W_{i}=\frac{V_{i}}{\sum_{i=1}^{n}V_{i}}$$

where $V_{i}$ is the coefficient of variation of the *i*th variable, $\sigma_{i}$ is the standard deviation of the *i*th variable, $\bar{x_{i}}$ is the average of the *i*th variable and $W_{i}$ is the weight.

The fluctuation coefficient of grain production is a reverse indicator, and the following positive treatment formula was used:

(3) $$y_{it}=\underset{1\leq i\leq n}{\max}\left\{x_{ij}\right\}-x_{ij}$$

The amount of pesticide application, fertilizer application, and plastic film use are moderate reverse indicators, for which the following positive treatment formula was used:

(4) $$y_{it}=\underset{1\leq i\leq n}{\max}|x_{ij}-\bar{x_{ij}}|-|x_{ij}-\bar{x_{ij}}|$$

The specific indicator definitions and weights are listed in Table 2.

(2) Independent variables. Following Zhang et al. [33], food consumption was divided into three categories: grain consumption, plant food consumption, and animal food consumption. The proportion of food consumption to total food consumption for the three types of food was calculated, in order to measure the structure of food consumption. Among them, grain consumption was measured as the sum of the annual per capita consumption of grain and edible oil by residents; plant-based food consumption was measured by the number of fresh vegetables consumed per capita annually; and animal food consumption was measured in terms of the total annual per capita consumption of meat, poultry, aquatic products, and eggs. Table 3 shows the food consumption structure of Chinese residents from 2015–2019, from which it can be seen that the total per capita food consumption of Chinese residents remained stable from 2015 to 2019, while the food consumption structure has undergone great changes. Among them, the decline in grain consumption was relatively large, from 134.50 kg in 2015 to 130.11 kg in 2019, and the proportion of food consumption also fell (from 45.56% to 43.84%). The consumption of animal food increased from 55.20 kg in 2015 to 61.93 kg in 2019, and its proportion in food consumption increased from 18.70% to 20.87%. There was little overall change in plant consumption.

(3) Mediating variables. The mediating variables included agricultural R&D and agricultural investment. As there was a lack of statistics on the full-time equivalent of agricultural R&D personnel and agricultural R&D expenditure in the existing statistical yearbook and database, we use the China national knowledge infrastructure patent database to manually count the number of agricultural patents in each province. The proportion of agricultural patents in various provinces with respect to the number of national patents was used to measure agricultural R&D. The reason for choosing this specific measure is that the relative difference can better reflect the proportional relationship with the population, and can better characterize the intensity of agricultural R&D between provinces [39]. For agricultural investment, we adopted the amount of investment in agriculture, forestry, animal husbandry, and fishery with respect to the fixed assets of rural households [36].

(4) Control variables. Provision of food security requires taking into account a number of factors that are independent of agriculture, but may significantly affect the level of food consumption [40]. Drawing on the research of Fang et al. [25], the transportation infrastructure, the degree of opening up, the industrial structure, population density, and per capita disposable income were selected as control variables. Among them, transportation infrastructure affects the logistics capacity of agricultural products, transportation infrastructure plays a pivotal role in ensuring food security [41]. The degree of opening up affects the circulation and trade capacity of grain, there is no doubt that the source and structure of imports affect food security. The industrial structure reflects the proportion of agriculture in the region in the regional economy, it reflects the level of agricultural production and processing in the region. The density of the population affects the total demand for food, and per capita disposable income affects the availability of food [42]. Some variables were transformed into logarithms, in order to avoid the impacts of unit inconsistencies and heteroscedasticity problems on parameter estimation. The variables are defined, and the descriptive statistical results are provided in Table 4.

### 3.2. Empirical Models

(1) Regression model. To test Hypothesis 1, an OLS regression model was constructed, in order to verify the impact of food consumption structure on food security.

(5) $$Y_{it}=\beta_{0}+\beta_{1}X_{it}+\beta_{2}Control_{it}+\varepsilon_{it}$$

where $Y_{it}$ represents the food security situation in the year *t* for the *i*th province and $X_{it}$ is the core explanatory variable, which specifically includes three aspects: grain consumption, plant food consumption, and animal food consumption. Considering the availability of data in conjunction with existing studies, the control variable $Control_{it}$ (including transportation infrastructure, degree of opening up, industrial structure, population density, and per capita disposable income) was added to the formula.

(2) Mediating effect model. To test Hypotheses 2 and 3—namely, to test the mediating effects of agricultural R&D and agricultural investment in the influence of food consumption structure on food security—a mediating effect test was carried out, with reference to the mediating effect test method and steps proposed by Judd et al., and the causal stepwise regression method was used [43]:

(6) $$M_{it}=\alpha_{0}+\alpha_{1}X_{it}+\alpha_{2}Control_{it}+\varepsilon_{it}$$

(7) $$Y_{it}=\gamma_{0}+\gamma_{1}X_{it}+\gamma_{2}M_{it}+\gamma_{3}Control_{it}+\varepsilon_{it}$$

where $Y_{it}$ represents the dependent variable in the mediating effect test, $X_{it}$ represents the independent variables for the mediating effect test including three aspects (grain, plant food, and animal food consumption), $M_{it}$ denotes the mediating variable (agricultural R&D and agricultural investment), and $Control_{it}$ denotes the control variable. The specific steps were as follows: First, use model (5) to test the impact of food consumption structure on food security. If the coefficient is significant, the variables are introduced into model (6) for mediating effect testing, in order to analyze the impacts of food consumption structure mediated by agricultural R&D and agricultural investment. Finally, if the coefficients are significant, the variables are brought into the model (7), and if the coefficient of $X_{it}$ in Formula (6) and the coefficient of $M_{it}$ in Formula (7) are both significant, the mediating effect exists.

## 4. Results and Analyses

### 4.1. Baseline Regression Results

Table 5 reports the regression results regarding the impact of food consumption structure on food security. From column (1), it can be seen that the coefficient of grain consumption was positive and significant at the 1% level. Therefore, Hypothesis 1 was verified, indicating that grain consumption positively affects food security. This conclusion differs from that of Chen and Lu [8], perhaps as the original allocation of availability factors was changed when grain consumption increased. When the allocation of resources is reasonable, production efficiency increases, and grain output increases. The results in column (2) show that the coefficient of plant food consumption was negative and significant at the 10% level, indicating that plant food consumption negatively affects food security. Thus, Hypothesis 2 was verified. From the regression results in column (3), the coefficient of animal food consumption was negative and significant at the 5% level, indicating that animal food consumption negatively affects food security. Thus, Hypothesis 3b was also verified. Assuming that Hypotheses 2 and 3 are related to the theoretical analysis in the preceding article, a possible explanation is that, due to the interaction between demand and supply, the relationship between food consumption structure and food security is complex. In view of the fact that, in this paper, we constructed the index system for food security considering the dimensions of stability, and sustainability in food production, with an increase in plant and animal food consumption, due to the scarcity of grain production factors, such as land resources, water resources, labor resources, and energy resources, once limited resources are invested in feed crops and cash crops, the planting space of food crops is inevitably compressed, thus affecting food security.

### 4.2. Results of Mediating Effect Analysis

Considering the test of the mediating effect of agricultural R&D. Table 6 reports the empirical results of food security impact mechanisms mediated by agricultural R&D. The results in column (1) of Table 6 indicate that grain consumption significantly affected agricultural R&D at the 10% level. The above findings confirm the previous theoretical assumption: an increase in grain consumption can optimize the coordination between sub-industries within the food industry system, thus improving the efficiency of resource utilization and enhancing the intensity of agricultural R&D. The results shown in Table 6, columns (2) and (3), indicate that plant food consumption not affected agricultural R&D, while animal-based food consumption positively affected agricultural R&D at the 1% level. In columns (4)–(6) of Table 6, which grain food, plant food, and animal food consumption as well as agricultural R&D into the model at the same time as independent variables affecting food security, the empirical results suggest that grain consumption, animal food consumption, and agricultural R&D were still significant after adding the agricultural R&D variable to the original equation. At the same time, the estimated values of the parameters were significant, indicating that the mediating effect of agricultural R&D exists, comprising a partial mediating effect. Among these, the proportion of the mediating effect to the total effect was (0.0148 × 6.1047)/2.0358 = 0.0443 and (0.2101 × 7.2114)/−3.6125 = 0.4194, respectively. This indicates that about 4.43% and 41.94% of the impact of grain consumption and animal food consumption on food security were realized through the intermediary role of agricultural R&D. Therefore, Hypothesis 4 was validated.

In terms of the test for the mediating effect of agricultural inputs, Table 7 examines the empirical results of food security impact mechanisms mediated by agricultural investment. The results shown in columns (1)–(3) of Table 7 indicate that grain consumption contributed to agricultural investment at the 10% level, while consumption of plant and animal food negatively affected agricultural investment at the 10% level, respectively. Columns (4)–(6) of Table 7 bring both agricultural investment and food consumption structures into the model as independent variables affecting food security levels. The results indicate that the coefficients of grain consumption and agricultural investment were significantly positive at the 1% level, indicating that agricultural investment had a mediating effect in the process of grain consumption affecting food security. Plant and animal food consumption also presented the same partial mediating effect. Among them, the proportion of the mediating effect to the total effect was (1.7191 × 0.3864)/1.4871 = 0.4466, (−0.4770 × 0.3994)/−0.8111 = 0.2348, and (−2.2397 × 0.3934)/−1.2817 = 0.6874, respectively. This indicates that about 44.66%, 23.48%, and 68.74% of the impacts of grain, plant food, and animal food consumption on food security were achieved through the intermediary role of agricultural investment. Thus, Hypothesis 5 was verified.

### 4.3. Robustness Test

In order to ensure the accuracy of the regression results, a stability test was carried out in the following ways. First, in order to determine the possible deviations in the measurement of the dependent variable, the metrics of the dependent variable and the original food security evaluation system were replaced. Referring to the five dimensions of quantity security, quality safety, ecological security, economic security, and resource security of Cui et al. [24], the main contents include: grain yield fluctuation coefficient, sown area and unit area yield, per capita grain occupancy, pesticide and fertilizer application, financial support for agriculture, grain disaster ratio, Engel coefficient, grain sales price index, unit cultivated land area, and water resources. These were used to replace the original food security evaluation system. The specific results are shown in Table 8, Columns (1)–(3). Notably, the conclusions of this study were basically consistent with those of the previous article. Second, the method of increasing the control variable was used to determine the estimation bias caused by possible missing variables. Considering that the more developed the economy and the higher the level of urbanization, the more complex the food consumption structure of a region, we added the economic development level (provincial GDP logarithm) and the urbanization level (urbanization rate) as control variables on the basis of the benchmark model [25]. In the results shown in Table 8, Columns (4)–(6), the coefficients of the main independent variables were basically consistent with those above, and the empirical results remained stable and reliable.

### 4.4. Heterogeneity Analysis

Analysis of urban–rural heterogeneity: Considering the differences in economic level, eating habits, and food access between urban and rural residents in China, the impact of grain consumption structure on food security may differ due to urban–rural heterogeneity. Table 9 reports the heterogeneity regression results for urban and rural sub-samples. Columns (1)–(3) are the results for the urban sample, while columns (4)–(6) report the results for the rural sample. Among them, the regression results in columns (1) and (4) show that both urban and rural samples presented a positive and significant impact on grain security; the regression results in columns (3) and (6) showed that animal food consumption had a negative and significant impact on food security, with a stronger effect on rural residents (coefficient of −2.1653) than urban residents (coefficient of −1.4015); and the regression results in columns (2) and (5) indicated that the effect of plant food consumption on food security was significantly negative in the urban sample alone and was negative in the rural sample, but not significant. The reason for this urban–rural difference may be explained by the fact that, on one hand, urban residents consume more vegetables than rural residents, occupy more food planting resources, and turn to the cultivation of cash crops such as vegetables, thus affecting the food supply; on the other hand, the plant food consumption of rural residents mainly comes from their household gardens or cultivation on their own ration land, making them mainly self-sufficient and, so, the associated impact on food security is relatively small.

Regional heterogeneity analysis: As there are great differences in endowment conditions and eating habits in different regions of China, in order to objectively reflect the possible regional differences in food security with respect to the food consumption structure, according to the division standards of the National Bureau of Statistics, the 31 provinces in the sample were divided into three regions—east, central, and west—and the differences and rationality of food security between different regions were examined. The OLS regression model was used for group testing, and the regression results regarding heterogeneity in different regions are shown in Table 10. The results indicated that the impact of grain consumption and animal food consumption on food security in the eastern region was consistent with the basic estimates of the whole sample, and the coefficient was significantly increased, possibly as the eastern region has a more developed economy and a higher degree of response to the transformation of the consumption structure. In the central region, plant food consumption and animal food consumption were significant at the 5% and 1% level, respectively, and grain consumption did not pass the significance test; perhaps because Heilongjiang, Henan, Jilin, Hunan, and Hubei, as important grain production bases in China, have a relatively large proportion of permanent cultivated land, allowing grain production to reach a relatively stable and saturated situation. As a result, the impact of changes in grain consumption on food security was relatively small. In the western region, the roles of grain consumption and plant-based food consumption on food security were significant at the 5% and 1% level, respectively, while the role of animal food consumption was negative, but not significant. The possible reason is that there are more pastoral areas in the western region—mainly animal husbandry—and the proportion of cattle and mutton consumption in the diet is relatively large. Therefore, the associated impact on the local grain supply was not obvious.

## 5. Conclusions and Suggestions

### 5.1. Conclusions

Studying the changes and trends in domestic food consumption is an important part of ensuring food security in China. Based on Marxist production and consumption theory, panel data from 31 provinces from 2015 to 2019 were used to verify the impact of grain, plant, and animal food consumption on food security, as well as the corresponding mechanisms. Our main conclusions are as follows: (1) Grain consumption had a positive and significant impact on food security, while plant and animal food consumption had negative and significant impacts on food security. This correlation remained true in stability tests, including those replacing the indicator measures of the interpreted variables and adding control variables. (2) The structure of food consumption has an impact on food security in two key ways: changing agricultural R&D and changing agricultural investment. (3) Heterogeneity analysis studies indicated that urban areas can strengthen the marginal impact of grain consumption and plant consumption on food security. Furthermore, compared with the central and western regions, grain consumption and animal food consumption in the eastern region had a stronger marginal impact on food security.

### 5.2. Suggestions

Based on the above research conclusions, the following four suggestions are proposed: First, the existing concept of food security should be adjusted, in order to adapt to the transformation of market demand. On the basis of stabilizing grain production, an ecological high-value grain industry system should be built around the changes in market consumption demand, thus promoting the high-quality development of the grain industry. The second aspect is paying attention to the impact of food consumption on supply factors and ensuring food security from the supply side. The connection of smart agriculture with green development and sustainable development highlights the support for typical application scenarios such as smart farms and smart agricultural machinery. The connection of agricultural research and development with the guarantee of food security and ecological security highlights the support for smart agricultural technology to save costs and increase the efficiency and marketization of small-scale agriculture. Furthermore, the establishment of a comprehensive rating system for key areas of agricultural investment should be conducted. In line with the results of the index calculation, the agricultural products focused on agricultural investment should be determined. The overall development goals and concrete production capacity goals should be set. After that, the main tasks and main investment channels should be clarified, so that the healthy and sustainable development of China’s agricultural investment can be promoted in an orderly manner. The third point involves improving the comprehensive grain production capacity in multiple dimensions. It is suggested that the planting structure and varieties are optimized and adjusted, adhering to the red line of cultivated land and ensuring the absolute safety of rations. It is also suggested that the construction of high-standard farmland in the main grain-producing areas is promoted, and high-quality development of the grain industry is achieved. The fourth suggestion is to correctly guide the food consumption and diet of residents, in order to balance and reduce food waste. High-quality meat, eggs, milk, and other agricultural products could be imported appropriately from overseas, in order to meet the need for green, personalized, low-carbon, and healthy dietary structures. By optimizing the dietary structure of residents, we may reduce food waste, thus alleviating pressure on resources and the environment and, in this way, ensuring China’s food security.

### 5.3. Theoretical Contribution

The marginal contributions of this paper are as follows: First, we enrich the literature on the economic consequences of food consumption. Previous discussions of food consumption have paid more attention to its characteristics, ecological footprint, and spatio-temporal evolution, such that research on the economic consequences of food consumption remains relatively lacking. This paper expands the research horizon of food consumption structure from the perspective of food consumption, as well as plant and animal food consumption. Second, this paper also enriches the relevant literature on the influencing factors of food security. Although previous research has focused on the supply level, in this paper, we consider the demand level, clarify the logical relationship and mechanism of food consumption structure on food security, and propose a feasible path to ensure food security at the macro level, providing a key reference for follow-up research. Third, we construct an improved index system for food security; namely, based on the relevant previous literature, a food security evaluation standard is constructed from the three dimensions of availability, stability, and sustainability, in order to provide an objective evaluation reference for subsequent empirical research.

### 5.4. Limitations

This study has some limitations that can inspire future research. First, there are limitations with respect to the sample data. Due to the difficulty of obtaining urban data in China, it cannot be realized to more accurately identify the causal relationship between Food Consumption Structure and Food Security at the city level. Hence, findings should be treated with caution. For future studies, a more accurate food security metric can be established to identify the causal relationship between the two. Second, the mechanistic analysis needs to be expanded. This study only analyzed agricultural R&D and investment play mediating roles in the impact of food consumption structure on food security, while it is obvious that this is not the only transmission mechanism. Future studies may consider other potential moderating variables. Third, on account of the significant difference in China’s dietary habits from other countries, the research findings may not be applied globally. Follow-up research can work with more data from developing countries to study issues related to differences in dietary habits in different countries.

## Figures and Tables

**Table 1:** Food security evaluation systems constructed by scholars.

Author and Year	Food Security Evaluation Index
FAO (2003) [16]	Four main dimensions of food security can be identified according to FAO. Physical availability of food, Economic and physical Access to food, Food utilization, Stability.
Cui et al. (2019) [24]	Five aspects: Quantity security, Quality security, Ecological security, Economic security, and resource security, Including fluctuation coefficients of grain production, Sown area and unit area output, Per capita grain Possession, Pesticide and fertilizer application, Financial support for agriculture, Proportion of grain disasters, Engel coefficient, Grain sales price index, Unit cultivated land area, and water resources.
Fang et al. (2020) [25]	Four dimensions: Supply ability, Availability, Stability, and sustainability, Including food share, Production fluctuation coefficient and disaster rate, Pesticide and fertilizer application, and mulch film use.
Mina and Kumar (2021) [26]	It includes food security outcomes of these activities related to availability and utilization of, and access to food as well as other socioeconomic and environmental factors.
Schindler et al. (2017) [23]	Environmental, Social, and economic analysis, Including crop diversity, Land fertility, Food intake diversity, Social eating habits, Grain yield, Dependence on foreign agricultural trade, and utilization of agricultural resources.
Jiang and Zhu (2021) [27]	Includes nutrition, Availability, Affordability, Stability, Sustainability, and Policy support. Including pesticide application, Fertilizer application, Density of population, Proportion of food disasters, Engel’s coefficient, Grain sown area, Road density, Food price volatility, Agricultural machinery power.
Jones et al. (2013) [28]	Malnutrition rates, Proportion of household food expenditure, Dietary structure index, Food price volatility index, Food adequacy, Cultural acceptability and food stability.
Headey et al. (2012) [29]	Personal nutrition, whether there are seasonal shortages, Dietary diversity, Calorie consumption, Malnutrition rates, Household food safety perceptions.
Zhou (2010) [30]	Adequacy of food supply includes Nutritional adequacy, Food safety and quality, Cultural acceptability. Stability of food supply and access includes Environmental stability, Social stability.
Upton et al. (2016) [14]	Four aspects: Accessibility, Sustainability, Availability, and stability, Including: Food supply and access, Household poverty rate, Total food supply, Food diversity score, Dietary diversity score, Food frequency score, and food consumption score.

**Table 2:** Food security evaluation index system.

Dimension	Indicator	Definition	Type	Wight
Availability	Grain production per unit area (tons/km^2^)	Total yield as a proportion of total sown area	Positive	0.07
	Grain sown area/%	The proportion of sown area in the provincial area	Positive	0.07
	Agricultural machinery power/10,000 kW	The total power of agricultural machinery power	Positive	0.10
	Fiscal Support for Agriculture/%	Agricultural expenditure as a proportion of total fiscal expenditure	Positive	0.17
	Food share per capita(kg/person)	The extent to which grain production meets the needs of the province	Positive	0.10
Stability	Fluctuation coefficient of food production/%	The difference between grain production and the average grain production accounts for the proportion of the average grain production	Negative	0.16
	Proportion of food disasters/%	The affected area as a proportion of the total sown area	Negative	0.15
Sustainability	Pesticide application (tons/thousand hectares)	The proportion of pesticide application to total sown area	Negative	0.05
	Fertilizer application (10,000 tons/1000 ha)	The proportion of chemical fertilizer application to total sown area	Negative	0.04
	Plastic film use (tons/1000 ha)	The proportion of plastic film used to total sown area	Negative	0.09

**Table 3:** Changes in the structure of food consumption of Chinese residents in 2015–2019.

Indicator	Year	Grain	Edible Oil	Vegetable	Meat	Poultry	Sea Food	Egg	Plant Food	Animal Food	Total
Food purchases per inhabitant (kg/person)	2015	134.50	10.61	94.88	26.20	8.36	11.18	9.46	94.88	55.20	295.18
	2016	132.82	10.61	96.95	26.10	9.13	11.44	9.66	96.95	56.32	296.70
	2017	130.12	10.42	96.11	26.67	8.89	11.46	10.01	96.11	57.03	293.68
	2018	127.23	9.64	93.01	29.52	9.00	11.39	9.70	93.01	59.61	289.49
	2019	130.11	9.47	95.21	26.91	10.80	13.57	10.66	95.21	61.93	296.72
Proportion of various types of food (%)	2015	45.56	3.59	32.14	8.87	2.83	3.78	3.20	32.14	18.70	100.00
	2016	44.76	3.57	32.67	8.79	3.07	3.85	3.26	32.67	18.94	100.00
	2017	44.30	3.54	32.72	9.08	3.02	3.90	3.41	32.72	19.41	100.00
	2018	43.94	3.33	32.12	10.19	3.10	3.93	3.35	32.12	20.59	100.00
	2019	43.84	3.19	32.08	9.06	3.63	4.57	3.59	32.08	20.87	100.00

**Table 4:** Definition of major variables and descriptive statistical analysis.

Type	Variables	Definition	Mean	SD	Min	Max
Dependent variable	Food Security	Calculated from food security assessment indicators and logarithm taken	6.6131	0.3796	5.7849	7.5180
Independent variables	Grain Consumption	Cereals, potatoes, legumes, and cooking oils account for the total food consumption	0.4906	0.0787	0.3716	0.8185
	Plant Food Consumption	Fresh vegetables account for the proportion of total food consumption	0.3239	0.0500	0.0686	0.4165
	Animal Food Consumption	Meat, poultry, aquatic products, and eggs account for the proportion of total food consumption	0.1855	0.0491	0.0954	0.3031
Mediating variables	Agricultural R&D	The number of patents for agriculture, forestry, animal husbandry and fishery in each province accounts for the proportion of the number of patents in the country	0.0323	0.0280	0.0004	0.1111
	Agricultural Investment	The amount of investment in agriculture, forestry, animal husbandry and fishery in the investment in agricultural fixed assets	0.6899	0.6320	0.0010	3.1540
Control variables	Transport Infrastructure	The mileage of transportation (railway, highway, waterway) line per square kilometer accounts for the proportion of the provincial area	1.0216	0.5952	0.0644	2.5290
	Degree of Opening Up	The total import and export volume of each province accounts for the proportion of regional GDP	0.2617	0.2954	0.0884	1.7881
	Industrial Structure	The output value of the primary industry accounts for the proportion of total output value	0.0921	0.0496	0.0030	0.2340
	Density of Population	The number of permanent residents in square kilometers (transformed into logarithms)	5.3352	1.4933	0.9701	8.2569
	Per Capita Disposable Income	Per capita disposable income (transformed into logarithms)	10.1021	0.3472	9.4136	11.1482

**Table 5:** Regression estimates the impact of food consumption structure on food security.

Variables	Food Security		
	(1)	(2)	(3)
Grain Consumption	2.1514 ***		
	(0.6967)		
Plant Food Consumption		−1.0027 *	
		(0.5906)	
Animal Food Consumption			−2.0972 **
			(0.9572)
Transport Infrastructure	0.1725 **	0.1672 *	0.2330 **
	(0.0854)	(0.0876)	(0.0903)
Degree of Opening Up	−0.8241 ***	−0.8853 ***	−0.6561 **
	(0.2865)	(0.2686)	(0.2615)
Industrial Structure	3.1391 **	1.7706	2.9294 **
	(1.2559)	(1.3967)	(1.1342)
Density of Population	0.1562 ***	0.1099 **	0.0822 **
	(0.0467)	(0.0458)	(0.0355)
Per Capita Disposal Income	0.4758 *	0.2442	0.3375
	(0.2793)	(0.2512)	(0.2465)
Year	Yes	Yes	Yes
Constant	−0.1795	3.9338	2.9402
	(2.9500)	(2.4291)	(2.3750)
Observations	155	155	155
F	4.5813	5.0824	4.8855
R-squared	0.3122	0.2778	0.2949

Note: Standard errors in parentheses. *, ** and *** indicate that the variable is significant at the level of 10%, 5% and 1%, respectively.

**Table 6:** The mediating role of agricultural R&D in the impact of food consumption structure on food security.

Variables	Agricultural R&D			Food Security		
	(1)	(2)	(3)	(4)	(5)	(6)
Grain Consumption	0.0148 *			2.0358 ***		
	(0.0082)			(0.6310)		
Plant Food Consumption		−0.1771			0.0989	
		(0.1561)			(0.6137)	
Animal Food Consumption			0.2101 ***			−3.6125 ***
			(0.0568)			(0.7812)
Agricultural R&D				6.1047 ***	6.2204 ***	7.2114 ***
				(0.8068)	(0.8469)	(0.8145)
Controls	Yes	Yes	Yes	Yes	Yes	Yes
Constant	0.0377	0.0021	0.2201	−0.4097	3.9207 *	1.3531
	(0.2161)	(0.1397)	(0.1347)	(2.8497)	(2.2184)	(2.2633)
Observations	155	155	155	155	155	155
F	9.1516	7.4222	7.9603	15.8394	15.9315	15.8829
R-squared	0.2340	0.2773	0.2790	0.4683	0.4307	0.5000

Note: Standard errors in parentheses. * and *** indicate that the variable is significant at the level of 10% and 1%, respectively.

**Table 7:** The mediating role of agricultural investment in the impact of food consumption structure on food security.

Variables	Agricultural Investment			Food Security		
	(1)	(2)	(3)	(4)	(5)	(6)
Grain Consumption	1.7191 *			1.4871 ***		
	(0.9678)			(0.4950)		
Plant Food Consumption		−0.4770 *			−0.8141 **	
		(0..2672)			(0.3913)	
Animal Food Consumption			−2.2397 *			−1.2817 *
			(1.2823)			(0.6719)
Agricultural Investment				0.3864 ***	0.3994 ***	0.3934 ***
				(0.0421)	(0.0439)	(0.0408)
Controls	Yes	Yes	Yes	Yes	Yes	Yes
Constant	−7.3181 *	−3.2932	−5.1165 *	2.6248	5.5690 ***	4.9610 ***
	(3.8137)	(3.0438)	(2.9745)	(1.8679)	(1.4250)	(1.4244)
Observations	155	155	155	155	155	155
F	5.1617	5.9858	6.8459	20.2931	20.1656	19.9212
R-squared	0.1786	0.1696	0.1779	0.6548	0.6445	0.6488

Note: Standard errors in parentheses. *, ** and *** indicate that the variable is significant at the level of 10%, 5% and 1%, respectively.

**Table 8:** Robustness Test results.

Variables	New Food Security			Food Security		
	(1)	(2)	(3)	(4)	(5)	(6)
Grain Consumption	0.5345 ***			2.5530 ***		
	(0.1842)			(0.5597)		
Plant Food Consumption		−0.2594 *			−0.5610 *	
		(0.1542)			(0.308)	
Animal Food Consumption			−1.2112 ***			−2.7694 ***
			(0.1786)			(0.5821)
Controls	Yes	Yes	Yes	Yes	Yes	Yes
Constant	−0.7143	0.5247	−0.4069	−1.3921	1.7584	−1.4251
	(0.6076)	(0.4320)	(0.4398)	(1.7137)	(1.6762)	(1.5993)
Observations	155	155	155	155	155	155
F	26.2815	23.9944	42.2838	29.1315	24.3017	31.2624
R-squared	0.6592	0.6360	0.7223	0.7149	0.6696	0.7114

Note: Standard errors in parentheses. * and *** indicate that the variable is significant at the level of 10% and 1%, respectively.

**Table 9:** Estimation of urban-rural heterogeneity regression.

Variables	Food Security					
	Urban			Rural		
	(1)	(2)	(3)	(4)	(5)	(6)
Grain Consumption	2.9744 ***			1.4830 **		
	(0.8226)			(0.6769)		
Plant Food Consumption		−1.7149 ***			−0.4062	
		(0.6554)			(0.6102)	
Animal Food Consumption			−1.4015 *			−2.1653 **
			(0.7540)			(0.9616)
Transport Infrastructure	0.2030 **	0.1498 *	0.2358 **	0.1935 **	0.1870 **	0.2360 **
	(0.0836)	(0.0871)	(0.0904)	(0.0875)	(0.0894)	(0.0915)
Degree of Opening Up	−0.6917 **	−0.8728 ***	−0.6964 ***	−0.8312 ***	−0.8453 ***	−0.6334 **
	(0.2650)	(0.2444)	(0.2540)	(0.2751)	(0.2737)	(0.2597)
Industrial Structure	3.2872 ***	1.6216	2.5035 **	2.7972 **	1.7447	3.0043 ***
	(1.1691)	(1.3796)	(1.1909)	(1.3321)	(1.4004)	(1.1162)
Density of Population	0.1399 ***	0.1164 ***	0.0732 **	0.1309 ***	0.0903 **	0.0795 **
	(0.0400)	(0.0429)	(0.0354)	(0.0480)	(0.0456)	(0.0354)
Per Capita Disposal Income	0.3195	0.1785	0.2500	0.3787	0.2101	0.3230
	(0.2429)	(0.2339)	(0.2385)	(0.2720)	(0.2550)	(0.2357)
Year	Yes	Yes	Yes	Yes	Yes	Yes
Constant	1.1043	4.7476 **	3.7221	0.9960	4.0260	2.8797
	(2.4355)	(2.3080)	(2.3314)	(2.9474)	(2.4841)	(2.2897)
Observations	155	155	155	155	155	155
F	5.3232	4.6994	4.7885	4.5773	5.3352	5.0978
R-squared	0.3203	0.2866	0.2846	0.2927	0.2715	0.2951

Note: Standard errors in parentheses. *, ** and *** indicate that the variable is significant at the level of 10%, 5% and 1%, respectively.

**Table 10:** Estimation of heterogeneity regression in different regions.

Variables	Food Security								
	The Eastern Region			The Central Region			The Western Region		
	(1)	(2)	(3)	(4)	(5)	(6)	(7)	(8)	(9)
Grain Consumption	5.2532 ***			1.7895			1.4885 **		
	(0.8311)			(2.2379)			(0.7308)		
Plant Food Consumption		0.8030			−3.2160 **			−3.4372 ***	
		(1.0026)			(1.4403)			(1.0645)	
Animal Food Consumption			−5.6761 ***			−9.9319 ***			−0.0311
			(0.8744)			(3.0811)			(1.2915)
Transport Infrastructure	0.8630 ***	1.0309 ***	1.1388 ***	0.5719 **	0.6145 **	0.8200 ***	−0.2610 ***	−0.4038***	−0.2766 **
	(0.1166)	(0.1616)	(0.1087)	(0.2696)	(0.2769)	(0.2881)	(0.0787)	(0.0793)	(0.1114)
Degree of Opening Up	0.0892	−0.1998	0.3911 **	−1.5214	−1.8134 **	−2.9642 ***	2.0351 **	1.7273 *	2.5015 **
	(0.1514)	(0.1647)	(0.1885)	(0.9668)	(0.8825)	(0.8259)	(0.9928)	(0.9753)	(1.0949)
Industrial Structure	−2.4575 ***	−5.8037 ***	−2.5312 ***	6.5548 ***	6.1533 ***	2.8425 **	5.6254 ***	6.2891 ***	4.0821 ***
	(0.7927)	(0.8429)	(0.8439)	(1.5080)	(1.1773)	(1.2657)	(1.1146)	(1.1472)	(0.9467)
Density of Population	−0.4122 ***	−0.6353 ***	−0.7241 ***	−0.0863	−0.1265	−0.5027**	0.1603 ***	0.2419 ***	0.1126 ***
	(0.1087)	(0.1719)	(0.1118)	(0.1565)	(0.1429)	(0.1915)	(0.0386)	(0.0543)	(0.0329)
Per Capita Disposal Income	−0.6473 ***	−1.0299 ***	−0.8148 ***	0.2149	−0.3887	−4.6538 ***	0.5696	1.2364 *	−0.2075
	(0.1815)	(0.1994)	(0.1445)	(1.2371)	(0.8395)	(1.4457)	(0.5773)	(0.7007)	(0.4600)
Year	Yes	Yes	Yes	Yes	Yes	Yes	Yes	Yes	Yes
Constant	12.4995 ***	20.1570 ***	19.2140 ***	3.3087	11.4800	53.1697 ***	−1.4250	−6.2823	7.2217
	(1.9602)	(1.6348)	(1.2345)	(13.7270)	(8.8331)	(14.6461)	(5.9356)	(6.5903)	(4.3367)
Observations	60	60	60	45	45	45	50	50	50
F	26.0232	27.7174	70.8286	4.2023	4.8764	6.5521	11.2196	10.1654	9.4065
R-squared	0.8460	0.7433	0.8707	0.5214	0.5629	0.6064	0.6786	0.7190	0.6483

Note: Standard errors in parentheses. *, ** and *** indicate that the variable is significant at the level of 10%, 5%, and 1%, respectively.

## Data Availability

Data are available upon request from the researchers who meet the eligibility criteria. Kindly contact the first author privately through e-mail.
